# Long-term eculizumab improves clinical outcomes in atypical hemolytic uremic syndrome

**DOI:** 10.1007/s00467-012-2276-8

**Published:** 2012-08-14

**Authors:** Ramon Vilalta, Enrique Lara, Alvaro Madrid, Sara Chocron, Marina Muñoz, Alex Casquero, Jose Nieto

**Affiliations:** Pediatric Nephrology Department, Hospital Universitari Vall d’Hebron, Pg. Vall d’Hebron 129-139, 08039 Barcelona, Spain

## Abstract

**Background:**

Atypical hemolytic uremic syndrome (aHUS) is a rare genetic disorder caused by chronic uncontrolled complement activation.

**Case-diagnosis/treatment:**

We present a 4-year-old girl with aHUS who had multiple severe clinical manifestations of thrombotic microangiopathy (TMA) including acute kidney injury, dilated cardiomyopathy, and cardiorespiratory arrest. She was managed with intensive plasma exchange and hemodialysis, which could not halt the progression of TMA. The initial single dose of eculizumab only temporarily improved the clinical symptoms of TMA. Sustained improvement of renal, hematological, and cardiac values were only achieved upon institution of chronic treatment with eculizumab. During long-term treatment with eculizumab (>2.5 years), she has had no further clinical manifestations of TMA, and required neither plasma exchange nor hemodialysis.

**Conclusion:**

Chronic eculizumab treatment was associated with control of complement-mediated TMA and sustained long-term improvement in renal and cardiac function.

## Background

Atypical hemolytic uremic syndrome (aHUS) is a rare genetic disorder caused by chronic uncontrolled complement activation [1, 2]. Although complement mutations have been found in 50–70 % of patients with aHUS, identification of a genetic mutation is not necessary for diagnosis or treatment initiation [3]. aHUS is characterized by systemic thrombotic microangiopathy (TMA) and multiple organ damage, which result in significant morbidity and mortality [4]. Despite supportive management, including plasma exchange/infusion (PE/PI), 33–40 % of patients progress to end-stage renal disease (ESRD) or die at the first clinical manifestation [1, 4, 5]. Thus, there remains a need for new therapeutic strategies [6].

Eculizumab is a humanized monoclonal antibody to terminal complement protein C5 that prevents activation of the terminal complement pathway by binding C5 and inhibiting generation of pro-inflammatory C5a and the lytic C5b-9 membrane-attack complex. It is licensed for treatment of paroxysmal nocturnal hemoglobinuria (PNH) and aHUS (Soliris® SmPC, Alexion Europe). We report on a severely ill child with aHUS treated with eculizumab. The patient was initially managed with intensive PE, but clinical manifestations of aHUS were observed, including laboratory evidence of TMA and organ damage. A single dose of eculizumab initially led to marked improvement in renal function, but the clinical manifestations of TMA were again seen after 6 weeks. Chronic eculizumab treatment was introduced to maintain complete blockade of complement activation, which correlated with clinical improvement (Soliris® SmPC).

## Case report

**Fig. 1:**
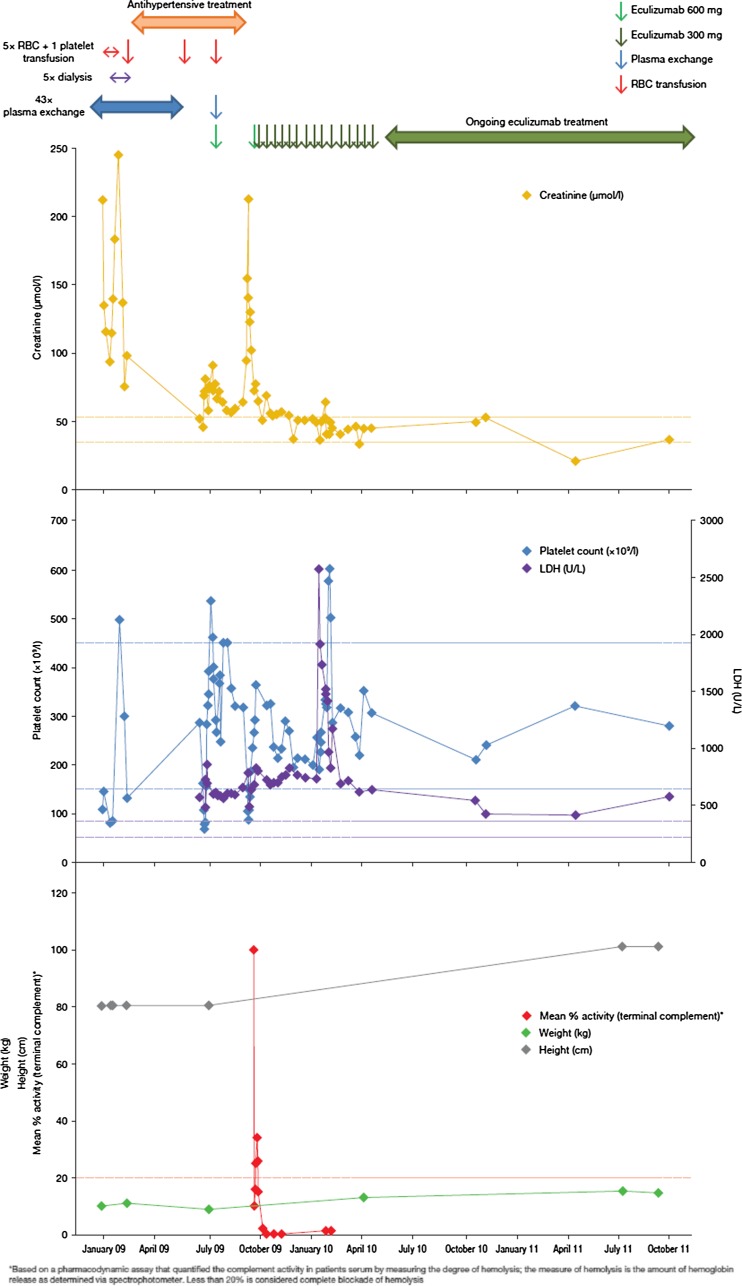
Response to eculizumab in a 1-year-old female with atypical hemolytic uremic syndrome (aHUS), as shown by creatinine levels, platelet counts, LDH, hemolysis assay of complement activation [unpublished data], and body weight and height. Initial plasma exchange and hemodialysis was started at the time of diagnosis, but the child experienced clinical manifestations of thrombotic microangiopathy (TMA) after approximately 7 weeks. Following cessation of plasma exchange, a single dose of eculizumab 600 mg was given (*green arrow*), which resulted in stabilization of renal and hematologic function. After approximately 8 weeks, clinical manifestations became apparent again as no complement inhibition was present, after which continuous eculizumab treatment was reinitiated (initial dose 600 mg, followed by 300 mg every 2–3 weeks). *Dashed lines* show the normal range for creatinine, platelets and LDH, and threshold for complete complement blockade

A severely ill 1-year-old Caucasian girl with a several-day history of vomiting and refusal to eat presented on 31 December 2008 with hemolytic anemia, thrombocytopenia, acute renal insufficiency, and intussusception. Schistocytes on peripheral blood film, elevated lactate dehydrogenase (LDH) 2674 U/l (normal range: 216–360 U/l), decreased platelets 108 × 10^9^/l (normal range: 150–350 × 10^9^/l), elevated creatinine 221 μmol/l (normal range: 35–55 μmol/l) and urea 234 mg/dl (normal range: 7–21 mg/dl), and reduced hemoglobin (Hb) 6.5 mg/dl (normal range: 10–14 mg/dl) and albumin 2.76 g/dl (normal range: 3.5–5.5 g/dl) were detected. Activity of ADAMTS-13 (Von Willebrand factor-cleaving protease) was within the normal range (83 %), thus excluding thrombotic thrombocytopenic purpura (TTP) [7]. Complement levels were within the normal range: C3, 94 mg/dl (normal range: 85–120 mg/dl); C4, 16 mg/dl (normal range: 15–40 mg/dl). As stool cultures for *Shigella*, *Salmonella*, *Yersinia*, and *Candida* were all negative, she was diagnosed with aHUS. Initial management comprised multiple blood transfusions, intensive plasma exchange (2–3 sessions per week), and hemodialysis. These interventions initially improved diuresis, the patient’s creatinine decreasing to a minimum of 94 μmol/l, but she continued to be hospitalized, to need repeated blood transfusions, have severe proteinuria (233 mg/dl), and suffer from hypertension (blood pressure increasing to 128/95 mmHg in the days following presentation) and edema. During the following weeks, she also suffered multiple catheter infections and had to maintain a nasogastric tube for feeding. Overall, the clinical status of the patient further deteriorated, presenting 17 February 2009 with acute renal insufficiency, severe anemia, macroscopic hematuria, proteinuria, arterial hypertension, and dilated cardiomyopathy. Laboratory values showed decreased platelet count (132 × 10^9^/l), an elevated LDH of 1740 U/l and continued anemia (Hb 7.1 mg/dl), and elevated serum creatinine (187 μmol/l). The child also experienced cardiorespiratory arrest requiring cardiopulmonary resuscitation and mechanical ventilation. Genetic analysis revealed a mutation in the C-terminal region of complement factor H (CFH) (a novel heterozygous mutation; 3355 G>A; Asp1119Asn; SCR19), with normal membrane cofactor protein (MCP) and Factor I, and no CFH autoantibodies. PE (fresh–frozen plasma, 2–3 times per week) was continued for the next 3 months, with blood transfusions given as required. The patient’s anti-hypertensive therapy (hydralazine, nifedipine, labetalol, and urapidil) was also continued (Fig. 1). She experienced pulmonary edema in March and again in May. Myocardial dysfunction was diagnosed and an ejection fraction of 32 % (normal values: 56–78 %) was measured in May. Due to concerns that this was caused by fluid overload from PE, PE and hemodialysis were tapered. This led to an improvement in renal function, but she continued to receive antihypertensive treatment and multiple blood transfusions (Fig. 1).

In July, when the patient presented again with cardiac dysfunction and an ejection fraction of 31 %, with moderately controlled blood pressure (100/85) and not managed with PE, the decision was made to initiate treatment with eculizumab; she received one 600-mg eculizumab infusion (at this time, the patient was vaccinated against *Neisseria meningococcus* serotypes A and Cm, and antibiotic prophylaxis with penicillin was provided). Eculizumab treatment led to normalization of renal and ventricular function, stabilization of hematologic parameters, and blockade of terminal complement activity (Fig. 1).

As the eculizumab dose was not repeated, and terminal complement blockade not maintained, TMA complications were seen again after 8 weeks (mid-September 2009), when the patient presented with renal insufficiency (creatinine, 141 μmol/l) and evidence of hemolysis and platelet consumption (LDH, 760 U/l; platelet count, 151 × 10^9^/l). Chronic eculizumab treatment was initiated following the recommended dosing by weight (5–10 kg) (Soliris® SmPC): initial dose 600 mg, followed by 300 mg every 2–3 weeks. At this time, the patient’s antihypertensive treatment was stopped. Creatinine started to improve prior to eculizumab initiation but levels normalized after 6 days of resuming eculizumab treatment (Fig. 1) and cardiac function improved. LDH levels stabilized but remained elevated (Fig. 1); the complement activity assay showed immediate, sustained blockade of terminal complement activation; and platelet counts remained within the normal range (Fig. 1). The elevated LDH was not the result of anemia, as Hb levels were normal (10 mg/dl) and no schistocytes were seen, with no further need for RBC transfusions.

At last follow-up visit (14 November 2011; >2.5 years since admission), the patient remained on chronic eculizumab treatment; no new clinical complications of TMA have occurred while receiving treatment, and no blood transfusion, plasma exchange, or hemodialysis has been necessary. Serum creatinine is 26.5 μmol/l, and serum protein 34 mg/dl, equivalent to 12 mg/m^2^/h. No further indication of hypertension or cardiac insufficiency has been noted and her ejection fraction has been normalized (64–70 %). The patient has gained 6 kg of weight and her overall health has improved and normalized entirely. She shows a completely normal development and full recovery of a good quality of life as reported by her parents.

During the >2.5-year period of chronic eculizumab treatment, measurements of complement activity have shown complete terminal complement blockade, which corroborate the clinical data of no manifestations of TMA (Fig. 1).

## Discussion

PE has been used historically for the management of aHUS [1], but in the present case, progressive renal deterioration and an increased need for blood transfusions was seen despite PE (2–3 sessions per week for 16 weeks). Progressive cardiac dysfunction was also detected. Blockade of the terminal complement protein C5 with eculizumab is a rational treatment for aHUS that directly targets chronic uncontrolled complement activity [6]. As demonstrated by the present case, a single infusion can have an immediate effect, but can only achieve terminal complement blockade for a short duration. A reduced dose or discontinuation of eculizumab treatment has previously been shown to lead to rapid deterioration in organ function [6, 8, 9]. Indeed, in the recent aHUS clinical trials with eculizumab, five of 18 patients who discontinued eculizumab experienced severe TMA complications following the missed eculizumab dose (Soliris® SmPC).

In this patient, following discontinuation of eculizumab, reinitiation of eculizumab therapy rapidly restored the clinical and biological parameters. However, several cases have been reported in literature where this has not been possible and reinitiation of eculizumab therapy following discontinuation cannot be counted on to salvage organ function [6]. This patient maintained an elevated LDH (a measure of tissue damage and/or hemolysis), although the level stabilized during eculizumab treatment. The elevated level may be the result of organ damage, as no further anemia and thrombocytopenia were detected. Complement activation can be further amplified by common triggers such as infection. In our patient, this manifested as a large, transient increase in LDH during a respiratory tract infection. However, despite this increase, there was no evidence of complement-mediated TMA during eculizumab treatment; renal function remained stable and hematologic function was rapidly normalized without a requirement for blood transfusions.

In contrast to PE, eculizumab treatment inhibits the underlying process of chronic uncontrolled complement activation. Thus, chronic eculizumab treatment permanently suppresses clinical manifestations of TMA. Cardiac function markedly improved in this patient during eculizumab treatment. The acute cardiac failure in February 2009 could have been caused by fluid overload due to PE and high BP. However, the persistent cardiomyopathy could not have been caused by elevated blood pressure or fluid overload alone as cardiomyopathy persisted well after PE had been terminated (April 2009) and blood pressure was fairly controlled, and did not improve until after chronic eculizumab therapy was initiated, thus suggesting TMA could be one of the causes. No histological studies of the myocardium were, however, conducted.

Long-term inhibition of chronic uncontrolled complement activation and complement-mediated TMA was accompanied by general improvement in the child’s clinical condition, including appropriate weight gain and a lack of subsequent events. The child has been treated for more than 2.5 years and treatment is well tolerated.

In conclusion, these findings are consistent with previous case reports of eculizumab in patients with aHUS, in which treatment was associated with control of complement-mediated TMA [6, 8, 9]. In addition, terminal complement blockade with eculizumab was directly correlated with sustained, long-term (>2.5 years) marked improvements in renal and cardiac function. Interruption of eculizumab treatment led to a rapid return of the clinical manifestations of TMA, but improvement and stabilization of organ function and hematological values was restored upon reinitiation of therapy in this case.
